# Constant plasma volume and colloid osmotic pressure after infusion of albumin 20%: A secondary analysis

**DOI:** 10.14814/phy2.70623

**Published:** 2025-10-22

**Authors:** Robert G. Hahn

**Affiliations:** ^1^ Research & Development Karolinska Institute at Danderyds Hospital (KIDS) Stockholm Sweden

**Keywords:** hemodilution, hyper‐oncotic albumin, infusion, pharmacokinetics

## Abstract

Aberrant elimination patterns after intravenous infusion of albumin 20% may predispose to persistent vascular overload or hyper‐oncoticity. In the present report, the frequency of deviating elimination patterns was studied by visual examination followed by calculations of the volume and albumin kinetics over 5 hours in 86 volunteers and clinical patients who received 3 mL/kg albumin 20% over 30 min (mean, 231 mL). Constant and virtually unchanged plasma volume expansion (i.e., steady state) developed after 21% of all infusions and developed most often during surgery (64%) but rarely in volunteers (3%). The subsequent kinetic analysis associated such steady state with slow capillary filtration (−27% vs. all others, *p* < 0.01). Steady state or a gradual increase of the plasma oncotic pressure occurred in 28% and was characterized by fast turnover of fluid, which taken together resulted in a dehydrating effect. By contrast, steady state for plasma albumin was associated with a 48% lower capillary leakage rate constant for albumin (*p* < 0.001) as compared to the other experiments. In conclusion, steady state for plasma volume and increasing colloid osmotic pressure after infusion of albumin 20% was more dependent on variations in fluid kinetics than on albumin kinetics.

## 1 INTRODUCTION

Intravenous infusion of albumin 20% is used to treat hypovolemia in surgical and intensive care patients. The changes in blood chemistry resulting from such infusions are well known and consist of the expansion of the plasma volume (PV) and increases in the plasma albumin concentration, colloid osmotic pressure (COP), and intravascular albumin mass.

These elevations usually subside over time at a rate that can be quantified by a half‐life. PV expansion has a half‐life of 5 h in volunteers (Zdolsek et al., 2020, 2022), 10 h in postoperative patients (Hasselgren et al., 2019), and 21 h during surgery (Gunnström et al., 2022). The corresponding half‐lives for the intravascular albumin mass have been between 6 and 10 h (Hasselgren et al., 2019; Zdolsek et al., 2020, 2022) although reported to be 20 h during surgery (Gunnström et al., 2022). Infusion‐induced PV expansion and the accompanying increase in albumin mass are then expected to decrease by 30–50% during a 5‐hour follow‐up period, except during surgery when the decrease would average 15%. However, the PV expansion and the albumin mass may show a deviating time course in individual subjects and occasionally remain unchanged (“steady state,” SS) or even increase, which might predispose to vascular overload. Another possibility is that SS with anuria and kidney failure develops if more 20% albumin or some other hyper‐oncotic fluid is administered (Ferraboli et al., 1997; Moran & Kapsner, 1987; Rozich & Paul, 1989). From a physiological point of view, a rise in COP would retain fluid in the glomeruli, but albumin 20% actually operates as a diuretic with three times more volume excreted than infused over a 5‐hour period (Gunnström et al., 2022; Hasselgren et al., 2019; Zdolsek et al., 2020, 2022). The increased diuresis makes the development of oliguria after treatment with hyper‐oncotic fluid, which has been reported in the literature, difficult to understand (Ferraboli et al., 1997; Moran & Kapsner, 1987; Rozich & Paul, 1989).

Little is known about the risk of vascular overload, but previous research has considered that albumin administration might induce renal failure. Shortgen et al. found that albumin 20% increased the occurrence of renal complications as compared to crystalloid fluid (Schortgen et al., 2008). Others do not support that view (Caironi et al., 2014; Jacob et al., 2008; Jakob, 2004; Wiedermann et al., 2010) but the clinician must still accept that a small risk of hyper‐oncotic oliguria/anuria exists when prescribing albumin 20%.

The aim of the present study was to identify early patterns in the body's handling of 20% albumin in volunteers and clinical patients that clearly deviate from the expected course. The considered patterns were post‐infusion SS or gradual increase of the plasma dilution, intravascular albumin mass, plasma COP, and plasma albumin concentration. Such aberrations may precede overt complications, such as fluid overload and oliguria/anuria, if aggravated by later and more liberal administration of hyper‐oncotic fluid. The hypothesis was that one or several of these patterns can be understood from the kinetic handling of fluid and albumin.

## 2 MATERIALS AND METHODS

### 2.1 Subjects

This report is a secondary analysis of data from four prospective non‐randomized clinical studies in which 3 mL/kg of albumin 20% (mean, 231 mL) was administered at a constant rate to 86 adult humans. The settings were the following: 41 healthy volunteers (Zdolsek et al., 2020, 2022), 15 post‐burn patients (Zdolsek et al., 2020), 15 postoperative patients (Hasselgren et al., 2019), and 15 patients studied during ongoing surgery with minor blood loss (Gunnström et al., 2022). The infusion time was 30 min, except in 12 volunteers where it was 2 h. Demographic and biochemical data for these groups are shown in Table 1.

**TABLE 1:** Demographic and biochemical data for the study groups.

Variable	Volunteers	Post‐burn	Surgery	Postoperative
*N*	41	15	15	15
Age (years)	30 ± 11	45 ± 15	46 ± 15	65 ± 13^a^
Body weight (kg)	74 ± 11	95 ± 17^a^	72 ± 16	73 ± 13
Females/males	18/23	3/12	10/5	2/13
Infused fluid volume (mL)	223 ± 34	281 ± 56	216 ± 48	218 ± 42
Plasma albumin (g/L), baseline	40 ± 2	24 ± 5^a^	37 ± 2	25 ± 5^a^
MAP (mmHg), baseline	82 ± 17	87 ± 1	65 ± 6	75 ± 8
C‐reactive protein (mg/L)	2 ± 3	86 (20–294)	<5^b^	61 (21–216)
Inflammation	No	Yes	No	Yes
Blood loss (mL)	140	140	340 (190–1340)	140
P‐creatinine (μmol/L)	78 ± 20	78 ± 13	71 ± 10	67 ± 11
Maximum plasma dilution (%)	15.3 ± 5.4	16.3 ± 6.0	13.6 ± 5.6	13.3 ± 4.9
Urine flow <0.5 mL/kg/h (*N*)	1	0	2	5
References	Zdolsek et al. (2020, 2022)	Zdolsek et al. (2020)	Gunnström et al. (2022)	Hasselgren et al. (2019)

*Note*: Data are the mean ± standard deviation or the median (range) depending on the distribution.

^a^ Significantly different from the other groups (Scheffé test *p* < 0.05).

^b^ At the end of surgery.

### 2.2 Inclusion and exclusion criteria

The four studies included fully conscious subjects aged 18–80 years in good health status [American Society of Anesthesiologists' (ASA) group I–II]. The volunteer group included subjects with no daily medication and blood hemoglobin (Hb) concentration of >100 g/L (Zdolsek et al., 2020, 2022). The post‐burn study recruited patients with a burn area >6% of the total body surface area (Zdolsek et al., 2020). The study of postoperative patients required a Hb concentration of >90 g/L (Hasselgren et al., 2019). The elective surgery patients were scheduled for operations lasting for >5 h but with expected minimal hemorrhage (Hasselgren et al., 2019).

### 2.3 Health conditions and treatments

The volunteers fasted from midnight but ingested one sandwich and drank one glass (200 mL) of clear liquid, 2 h before any blood sampling was started between 7.00 a.m. and 9.00 a.m.

The post‐burn patients were studied in the morning approximately 1 week after the injury. They were hemodynamically stable and had fasted overnight but were allowed to ingest one sandwich and drink one glass (250 mL) of liquid 1.5 h prior to the experiment.

The patients undergoing elective surgery with minor blood loss had fasted from midnight and were premedicated with 1 g of oral paracetamol. The surgeries included correction of pro‐ or retrognathia and breast reconstruction after ablatio. General anesthesia without additional epidural analgesia was used. Glucose 2.5% with electrolytes (0.5 mL/kg/h) was used to compensate for evaporation. A low‐dose infusion of norepinephrine (mean rate 2.6 μg/min) was given intermittently.

The study of the postoperative patients began at 6:30 a.m. after having undergone open abdominal surgery for cancer during the preceding day. The hemorrhage averaged 700 mL, and the operations had lasted 5.9 h. No colloid was given after midnight before the study. Hemodynamic stability was confirmed before the albumin infusion was started.

The post‐burn and postoperative patients were considered “inflammatory” as their plasma C‐reactive protein concentrations were elevated (60–90 mg/L) (Table 1).

### 2.4 Data collection and analysis

Blood (10 mL) was withdrawn at 15 occasions over 5 h after starting the infusion (0, 10, 20, 30, 40, 50, 60, 75, 90, 120, 150, 180, 210, 240, and 300 min). All sampling was performed with the subject lying in the flat recumbent position. The total volume was 140 mL.

Blood Hb, hematocrit (Hct), and plasma albumin were measured in the hospital's routine blood chemistry laboratory with coefficients of variation of 1%, 1%, and 2%, respectively, based on duplicate sampling at baseline.

An Osmomat 050 (Gonotec, Berlin, Germany) was used to measure plasma COP with a coefficient of variation of 2%.

Urine output was collected at 60 min and 300 min but also when urgency appeared. Subjects voided in the recumbent position.

The mean arterial pressure (MAP) was measured along with each blood sampling using an invasive or non‐invasive hemodynamic monitor.

The anthropometric PV was calculated according to Nadler et al. (Nadler et al., 1962) based on length, body weight, and sex.

### 2.5 Visual examination of curves

Printouts of the plasma dilution, COP, and albumin as well as the plasma albumin concentration corrected for dilution (=albumin mass) over a 5‐h period were judged in a single‐blind fashion to show either a slow but **gradual decrease** (normal), **no consistent change** (“steady state,” SS), or an **increase**. The use of the “albumin mass” as a variable is due to the fact that fluid‐induced dilution makes plasma albumin provide an erroneous figure for the amount of albumin that is present in the circulation.

A regression plot was made for each these variables between 30 min (end of infusion) and 300 min (end of experiment). “Normal” implied that the correlation coefficient for the regression line had a negative sign, “steady state” that the regression showed a horizontal (or close to horizontal) line, while “increase” implied that the correlation coefficient had a positive sign. In most cases, the “normal” curves decreased by more than 25% during the 5‐h period, but some curves could show and increasing or unchanged values during the first 2 h post‐infusion whereafter a clearly gradual decrease was initiated. These curves were graded as “normal” even if the total change was less than 25% (usually about 15%). The gradual decrease could also occur very slowly but was then deemed “normal” if the decrease was consistent and the data points were assembled closely around the regression line.

### 2.6 Calculations

Plasma dilution served as the index of PV expansion and was obtained as [(Hb_0_/Hb_t_) – 1]/(1– hematocrit_0_) where Hb_0_ denotes baseline and Hb_t_ the value at a later time. A minor correction was implemented to correct the dilution for blood sampling (150 mL per experiment) and other blood losses, if any (Löffel et al., 2021).

Population kinetic analysis was performed based on the measured urine output and frequent measurements of plasma dilution. The kinetic model quantified three flow rates over time: oncotic fluid shift, capillary leakage of fluid, and urine flow.

A kinetic analysis was also performed based on the plasma albumin concentration after correction for dilution; this variable represents the plasma albumin that would occur if no PV expansion had been induced. For simplicity, this variable is called “albumin mass.” This model quantified the size of a single body fluid volume occupied by the infused albumin mass and the rate of the transcapillary leakage of these molecules.

### 2.7 Kinetic analyses

The kinetic analysis of volume expansion implied that a one‐volume model with two administration routes (infusion and oncotic‐driven fluid shift) and two elimination routes (capillary leakage and urine flow) was fitted to all data on plasma dilution and urine output from all 86 experiments on a single occasion.

The kinetic analysis of the albumin mass was based on fitting a one‐volume model with a single administration route (the infused albumin) and one elimination route (net capillary leakage of albumin). The input data were the increase in plasma albumin since the infusion was initiated, corrected for plasma dilution; hence, [(albumin_1_ – albumin_0_) * (1 + plasma dilution)].

The analyses were performed using the Phoenix software for nonlinear mixed effects, version 8.3.4 (Pharsight, St. Louis, MO), and the First Order Conditional Estimation Extended Least‐Squares search routine. The initial curve‐fitting was followed by covariate analysis, which implied that a parameter could be modified due to the characteristics of one study group or a variable. Sensitivity analysis was performed using the “Profile” function in Phoenix.

A detailed explanation of the kinetic models and the analytical process is given in File S1.

### 2.8 Statistics

Data showing a normal distribution were reported as the mean ± standard deviation (SD) and differences between groups evaluated by one‐way ANOVA followed by the Scheffé test for post hoc analysis. Data showing a skewed distribution were given as the median (25th–75th percentiles) and comparisons made by Mann–Whitney's test. Incidences were compared using the chi‐squared test. Relationships between continuous variables were examined by linear regression. The statistical package used was SPSS v. 29.0.2.0 for Mac (IBM Corp., Armonk, NY). *p* < 0.05 was significant.

Statistics of the incidence of deviating patterns was based on the combined incidences of SS and increasing variable levels to provide sufficient analytical power.

Kinetic parameters are reported as the best estimate and 95% confidence interval (CI) as given by the Phoenix program. A covariate was statistically significant at *p* < 0.05 if its inclusion decreased −2 log likelihood for the model by >3.84 points.

## 3 RESULTS

### 3.1 Visual inspection

**FIGURE 1:**
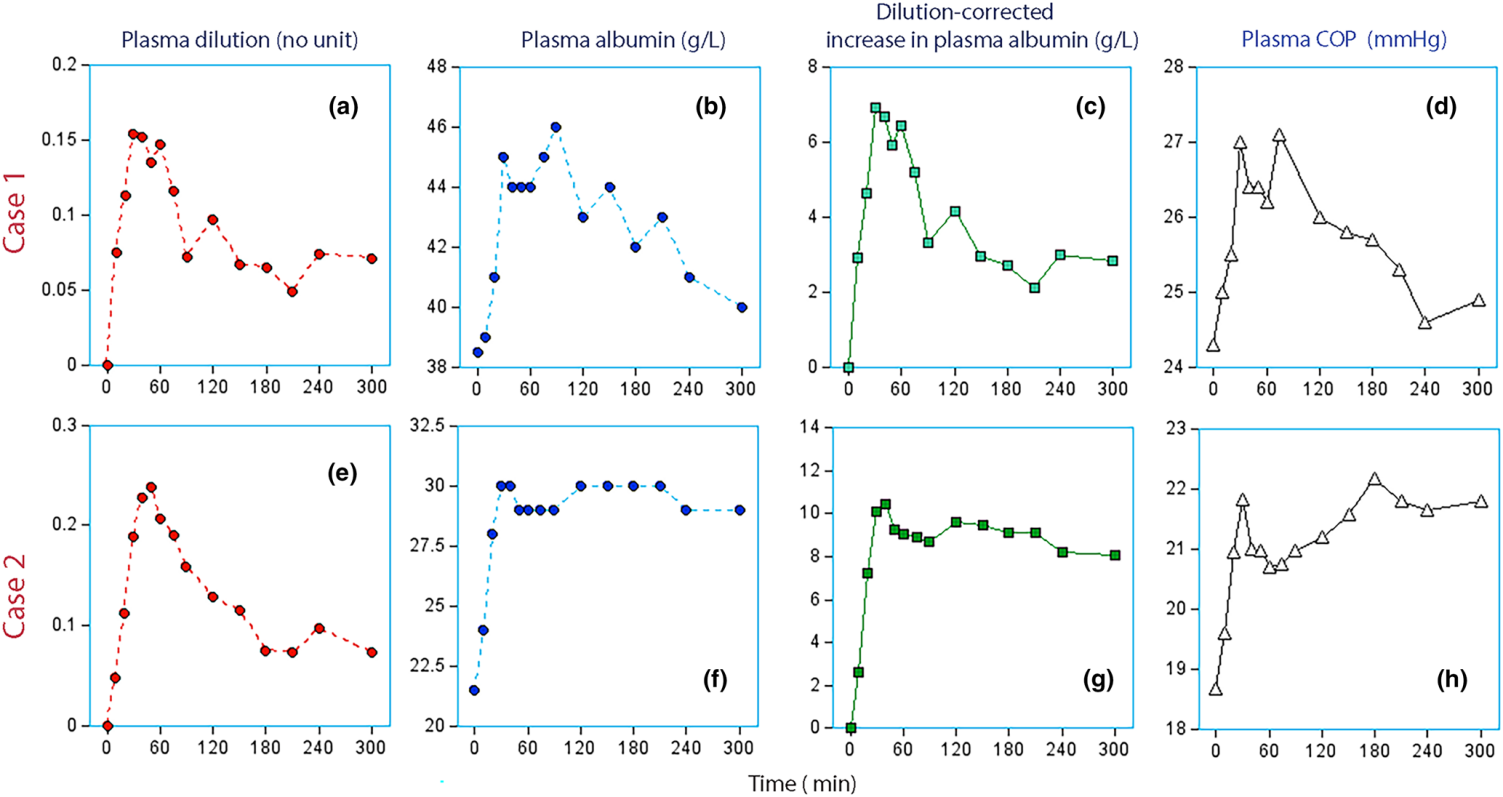
Individual curves. Top row: Plasma dilution, plasma albumin, increase in intravascular albumin mass (dilution‐corrected plasma albumin), and the plasma colloid osmotic pressure (P‐COP) in one healthy volunteer for 5 h starting with a 30‐min infusion of 3 mL/kg of 20% albumin. Normal curves showing a slow decrease. Bottom row: The same variables in a post‐burn patient judged to show steady state in plasma albumin and increase in P‐COP. Note rapid decrease of the plasma dilution.

A smooth decrease ideally occurred in all four variables (Figure 1, top row), but isolated SS for plasma albumin and plasma COP was also encountered (Figure 1, bottom row).

**FIGURE 2:**
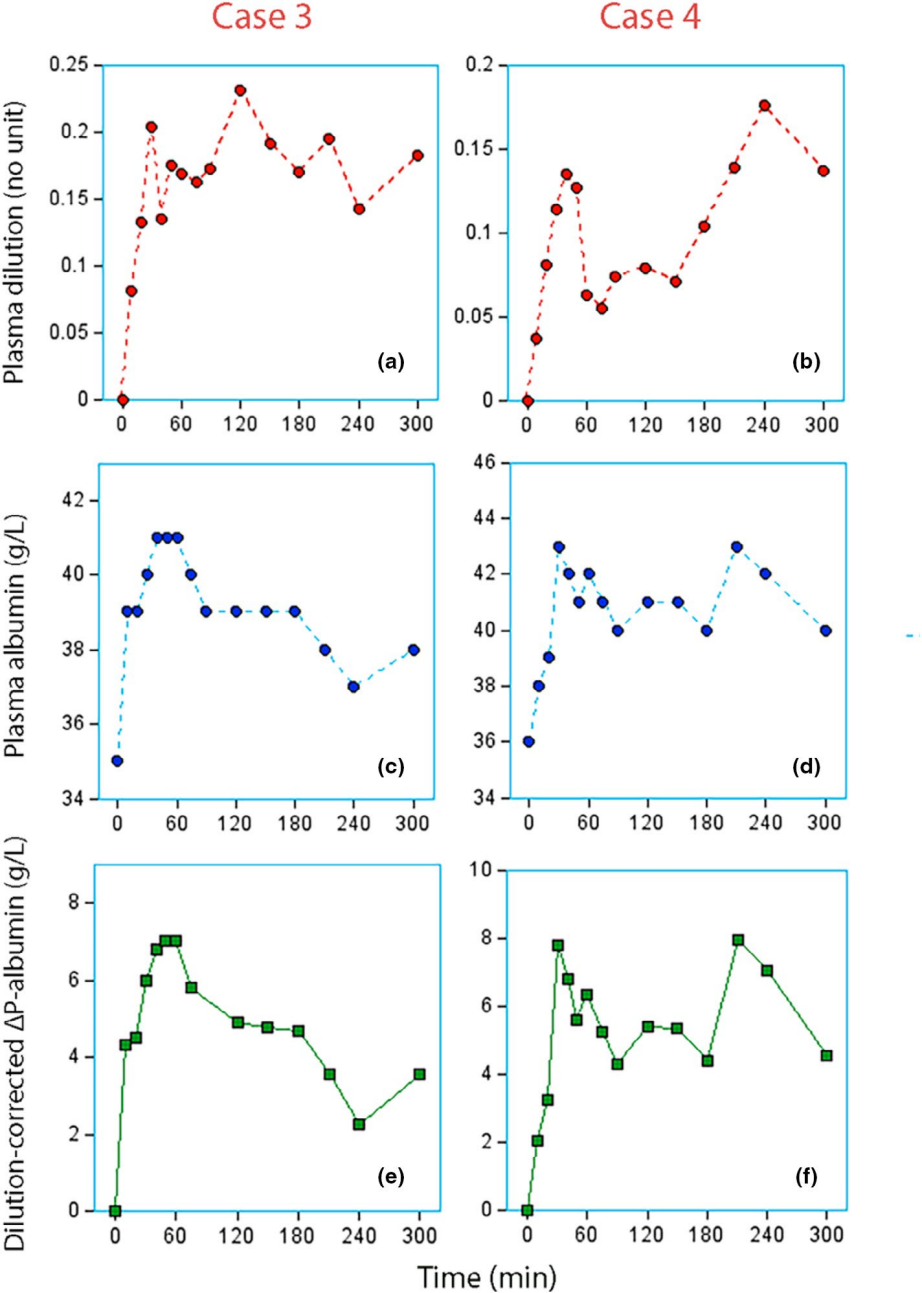
Individual curves. Left column: Patient undergoing surgery showing steady state in plasma dilution while the capillary leakage of albumin was deemed to be normal. Right column: Patient undergoing surgery who had a rapid initial capillary leakage of fluid and albumin and then probably a boost of albumin‐rich lymph at 180 min. This is a phenomenon called “interstitial washdown” and is characterized by increased plasma dilution at the same time as plasma albumin increases.

Another pattern was that the dilution hardly changed over time while plasma albumin decreased as expected (Figure 2, left column). A variant is shown in Figure 2, right column, where a boost of albumin‐containing lymph probably entered the circulation after 3 h.

Suspected deviating development of at least one of the four variables was found in 63% of the infusion experiments. For each variable, the incidence was approximately 30% (Table 2). Eighteen of the total number of 315 curves could not be classified due to unclear trends.

**TABLE 2:** Distribution of normal and deviating patterns in biochemical variables during 5 h after administration of 3 mL/kg of 20% albumin in 86 adult humans.

	Plasma dilution	Plasma COP^a^	Plasma albumin	Intravascular albumin mass
Gradual decrease	67	39	56	67
Steady state (SS)	9	8	21	10
Increase	6	8	2	5

^a^ Plasma COP was not available for 30 subjects.

### 3.2 Risk factors and inter‐correlations

The plasma creatinine concentration was higher, and MAP was lower, in those who developed SS for plasma dilution compared with those who showed a gradual decrease in this variable (84 ± 14 vs. 73 ± 15 μmol/L; *p* < 0.01, and 78 ± 17 vs. 87 ± 12 mmHg; *p* < 0.01, respectively). Subjects who developed SS for the intravascular albumin mass also had lower MAP at baseline (79 ± 17 vs. 86 ± 12 mmHg; *p* < 0.04). No demographic trait distinguished participants who showed SS for plasma albumin or COP from the others.

As expected, SS for plasma dilution and albumin mass often occurred together (86%, *p* < 0.001), and the normal decrease also showed a high degree of concordance (97%).

SS for plasma albumin and plasma COP also occurred together, but the concordance was weaker (71%/76%; *p* < 0.01). By contrast, SS for plasma dilution and plasma albumin were not significantly associated.

### 3.3 Special settings

SS for plasma dilution occurred in only one volunteer (3%), while the incidence was 64% during surgery (both comparisons yielded *p* < 0.001 vs. all others). Therefore, volunteers also had a low incidence of SS for intravascular albumin mass (20% vs. 54%; *p* < 0.02), while those undergoing surgery had an unusually high occurrence of SS for intravascular albumin mass (53% vs. 6%; *p* < 0.001). The postoperative and post‐burn patients had no deviating patterns that distinguished them from the other groups.

### 3.4 Kinetics of fluid and albumin

**FIGURE 3:**
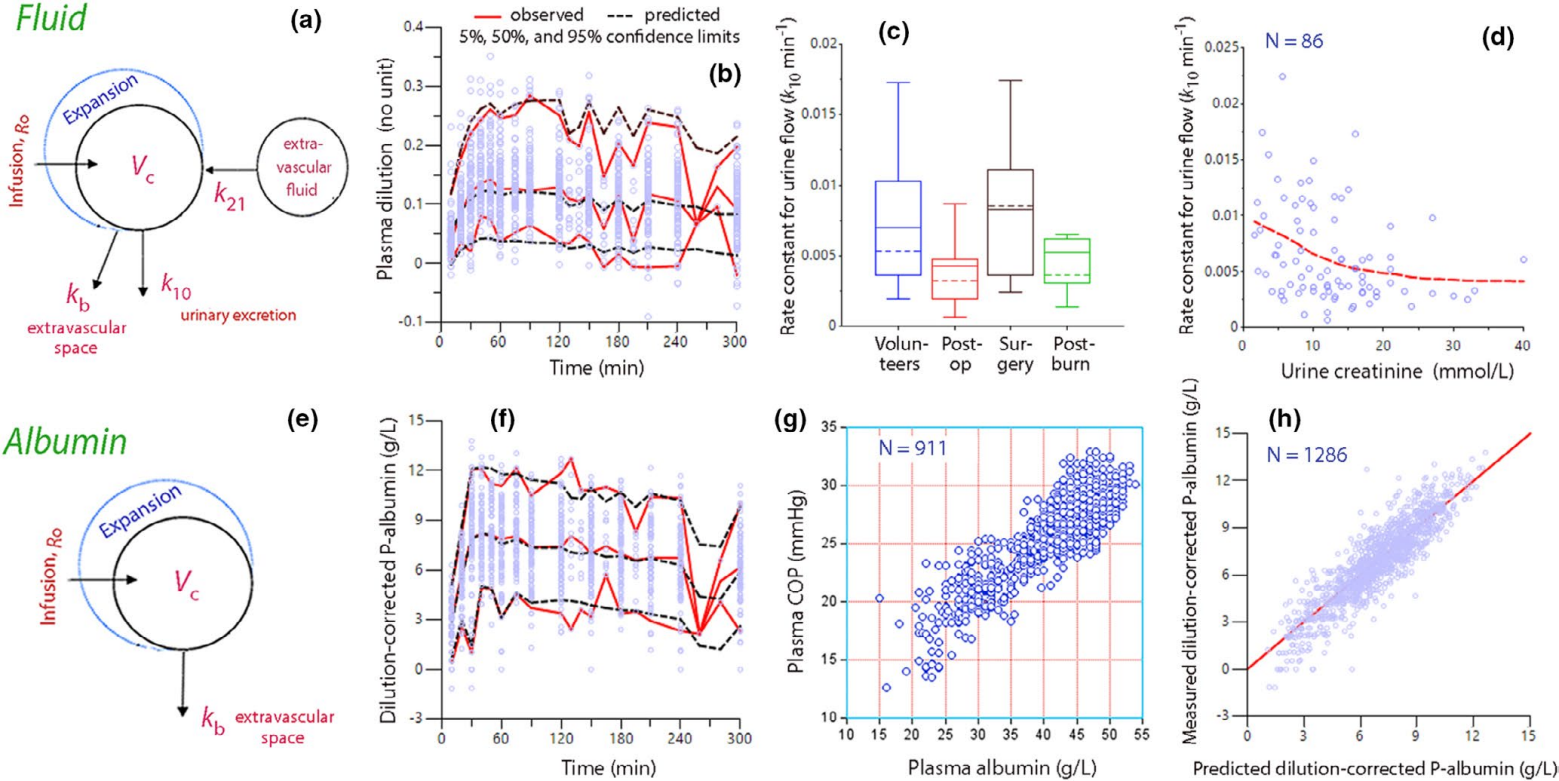
Kinetic models for fluid and albumin kinetics. (a) Schematic drawing of the kinetic model used to analyze the fluid volume distribution. (b) Predictive check for the fluid volume kinetics based on 1000 simulations. Close agreement between the observed and model‐predicted confidence intervals implicates that the model works well. (c) Boxplots showing the rate constant for urine flow (*k*_10_) in volunteers and in three special settings. (d) Covariance between the urine creatinine concentration before the infusions started and the rate constant for urine flow during the experiment. (e) Schematic drawing of the kinetic model used to analyze the infused albumin mass. (f) Predictive check for the infused albumin mass based on 1000 simulations. (g) Relationship between the plasma albumin concentration and the colloid osmotic pressure (COP) of the plasma. All measurements in the 86 experiments are shown. (h) Model‐predicted versus the measured dilution‐corrected plasma albumin concentration (i.e., the albumin mass).

**FIGURE 4:**
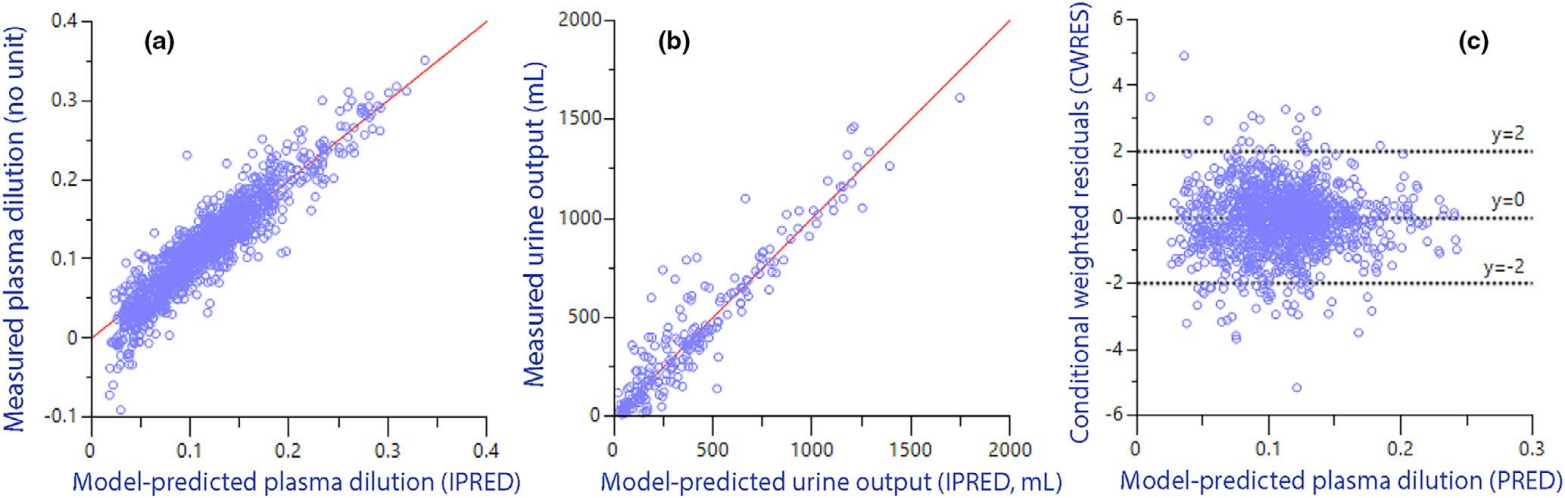
Performance measures for the analysis of fluid volume kinetics. (a) Relationship between measured plasma dilution and the predicted ones when the covariates have been considered (IPRED). Random distribution around the red solid line of unity indicates a good goodness‐of‐fit. (b) Same plot but showing the urine output. (c) Conditional weighted residuals (CWRES) versus the predicted plasma dilution without consideration taken of covariates (base model). Few observations outside 3 standard deviations suggests good model specification.

Fluid kinetic analysis was based on the measurements of plasma dilution (1286 data points) and urine output (210 collections). The model is illustrated in Figure 3a and performance measures are in Figure 3b and Figure 4.

The covariance analysis suggested that high plasma COP operated as a driving force for flow by accelerating capillary leakage and absorption of fluid from tissue (Table 3).

**TABLE 3:** Population kinetic parameters for the infused fluid volume in the final model. Shown are the typical values (tv) for the fixed parameters in the group, followed by individual‐specific covariates.

Kinetic parameter	Covariate	Covariate model	Best estimate	95% CI	CV%	–2 LL
tv*V*_c_ (L)			3.94	3.35–4.52	7.6	
tv*k*_10_ (10^−3^ min^−1^)			5.75	4.88–6.62	7.7	
tv*k*_21_ (10^−3^ min^−1^)			1.86	1.52–2.20	9.3	
tv*k*_b_ (10^−3^ min^−1^)			39.2	33.3–45.1	7.7	−1870
*k*_10_	Inflammation	Exponential	−0.31	−0.40 to −0.23	−13.5	−1945
*V*_c_	Female sex	Exponential	−0.31	−0.41 to −0.21	−16.9	−1996
*k*_10_	U‐creatinine	Power	−0.30	−0.39 to −0.21	−15.4	−2005
*V*_c_	Change COP	Linear	0.090	0.073–0.108	9.8	−2012
*k*_b_	Postoperative	Exponential	−0.18	−0.23 to −0.13	−13.8	−2018
*k*_10_	Postoperative	Exponential	−0.36	−0.47 to −0.26	−14.4	−2023
*k*_b_	Change COP	Linear^a^	0.36	0.31–0.41	6.5	–
*k*_21_	Change COP	Linear	0.34	0.30–0.38	5.6	−2093
Full block model	All the above					−2115

*Note*: *V*_c_ = conversion factor between volume and plasma dilution without correction for covariates. Rate constants describing flows: *k*_b_ = capillary leakage; *k*_10_ = urine flow; *k*_21_ = absorption of extravascular fluid. tv = typical value for the group. CI = confidence interval. CV% = coefficient of variation (inter‐individual). LL = log likelihood for the model during development. Decrease by >3.84 points = *p* < 0.05. U‐creatinine, mean = 12.6 mmol/L. Plasma colloid osmotic pressure (COP), mean change = 2.15 mmHg.

^a^ When the change in COP was tested as covariate to *k*_b_ and *k*_21_ one by one, −2 LL decreased by approximately 2.5 points, but an impressive decrease occurred when they were both tested simultaneously.

The urine flow was lower postoperatively and when the urine creatinine concentration had been high before the infusion was initiated (Figure 3c,d).

The *albumin kinetics* was analyzed based on the intravascular albumin mass (Figure 3e). The parameter estimates are shown in Table 4 and performance measures in Figure 3f–h.

**TABLE 4:** Population kinetic parameters for the infused albumin mass in the final model. Shown are the typical values (tv) for the fixed parameters in the group, followed by individual‐specific covariates.

Kinetic parameter	Covariate	Covariate model	Best estimate	95% CI	CV%	−2 LL
tv*V*_c_ (L)			5.75	5.46–6.04	2.6	
tv*k*_b_ (10^−4^ min^−1^)			8.31	6.65–9.98	10.2	4185
*V*_c_	Postoperative	Exponential	−0.033	−0.044 to −0.023	−15.6	4176
*V*_c_	Body weight	Power	1.48	1.25–1.70	7.7	4146
*V*_c_	Body mass index	Power	−0.89	−1.11 to −0.67	−12.5	4128
*V*_c_	Urine volume	Power	−0.095	−0.124 to −0.066	−15.5	4120
*V*_c_	Sex	Exponential	0.61	0.41–0.81	16.7	4106
*V*_c_	Inflammation	Exponential	−0.14	−0.20 to −0.087	−20.3	4100
*k*_b_	Body mass index	Power	−1.00	−1.26 to −0.73	−13.3	4095

*Note*: The full block model did not significantly improve the curve fit. Body weight, mean = 77 kg; body mass index, mean = 25.5 kg/m^2^; urine volume, mean of each subjects = 611 mL.

A sensitivity analysis of the fluid and albumin mass parameters showed minimal changes of −2 LL in response to perturbations of 20% and 50% (File S1).

The anthropometric size of the PV was 3.02 ± 0.61 L, and the size of the central volume (*V*_c_), based on post hoc data, that is, when the covariate effects had been considered, was 3.04 ± 1.25 L for fluid and 5.59 ± 1.59 L for the albumin mass.

### 3.5 Kinetics of steady state

SS for plasma dilution was associated with a 27% lower rate constant for the capillary leakage of fluid (*k*_b_) compared with the experiments that showed a gradual decrease in the dilution (Mann–Whitney's test *p* < 0.01) (Table 5).

**TABLE 5:** Kinetic parameters for curves showing steady state (SS) or increasing levels during the follow‐up time of 5 h versus those who did not develop SS for the same variable.

	Fluid volume kinetics			Albumin mass kinetics	
	*V* (L)	*k*_10_ (10^−3^, min^−1^)	*k*_b_ (10^−3^, min^−1^)	*V* (L)	*k*_b_ (10^−3^, min^−1^)
Plasma dilution					
SS	2.73 (2.23–3.86)	3.65 (3.16–8.18)	10.0 (8.4–13.8)**	5.41 (4.65–6.25)	1.08 (0.70–1.94)
Not SS	2.60 (2.16–3.87)	4.34 (3.66–7.76)	13.7 (10.9–17.0)	5.43 (4.28–6.44)	1.20 (0.82–1.68)
Albumin mass					
SS	2.73 (2.12–3.86)	3.54 (2.59–7.75)	9.34 (8.44–14.2)*	5.41 (4.28–6.44)	0.93 (0.63–1.40)
Not SS	2.74 (2.20–3.97)	4.03 (2.66–7.52)	13.4 (10.6–17.3)	5.43 (4.29–6.44)	1.22 (0.82–1.75)
Plasma COP					
SS	2.37 (2.07–3.98)	5.17 (3.46–10.3)*	14.0 (13.1–15.2)	5.82 (4.77–7.06)	0.84 (0.77–1.22)
Not SS	2.99 (2.34–4.08)	3.58 (2.58–5.32)	13.1 (8.95–16.2)	5.72 (4.70–6.71)	1.17 (0.72–1.68)
Plasma albumin					
SS	2.11 (1.41–3.83)	3.46 (2.44–8.96)	14.1 (11.2–15.4)	5.75 (4.68–6.40)	0.73 (0.62–0.98)***
Not SS	2.73 (2.26–3.64)	4.49 (3.12–7.79)	11.5 (9.28–14.3)	5.15 (4.09–6.09)	1.41 (1.13–2.09)

*Note*: Values for *k*_21_ are not shown as they showed limited variation at 0.68 × 10^−3^ min^−1^ within the 25th‐75th percentile range. Significance levels: *p < 0.05, **p< 0.01; ***p< 0.001 by Mann–Whitney's test.

Similarly, curves showing SS for albumin mass were characterized by a 30% lower value of *k*_b_ for fluid (*p* < 0.03).

SS for plasma COP was associated with a 44% higher rate constant for urine flow (*k*_10_) than the other experiments (*p* < 0.04).

SS for plasma albumin was associated with a 48% lower capillary leakage rate of the intravascular albumin mass (*p* < 0.001).

SS or increasing level of **any** of the four studied variables was associated with a 40% lower capillary leakage rate of the intravascular albumin mass (*p* < 0.003) and a slower capillary leakage rate of fluid (−20%; *p* < 0.02).

### 3.6 Simulations

**FIGURE 5:**
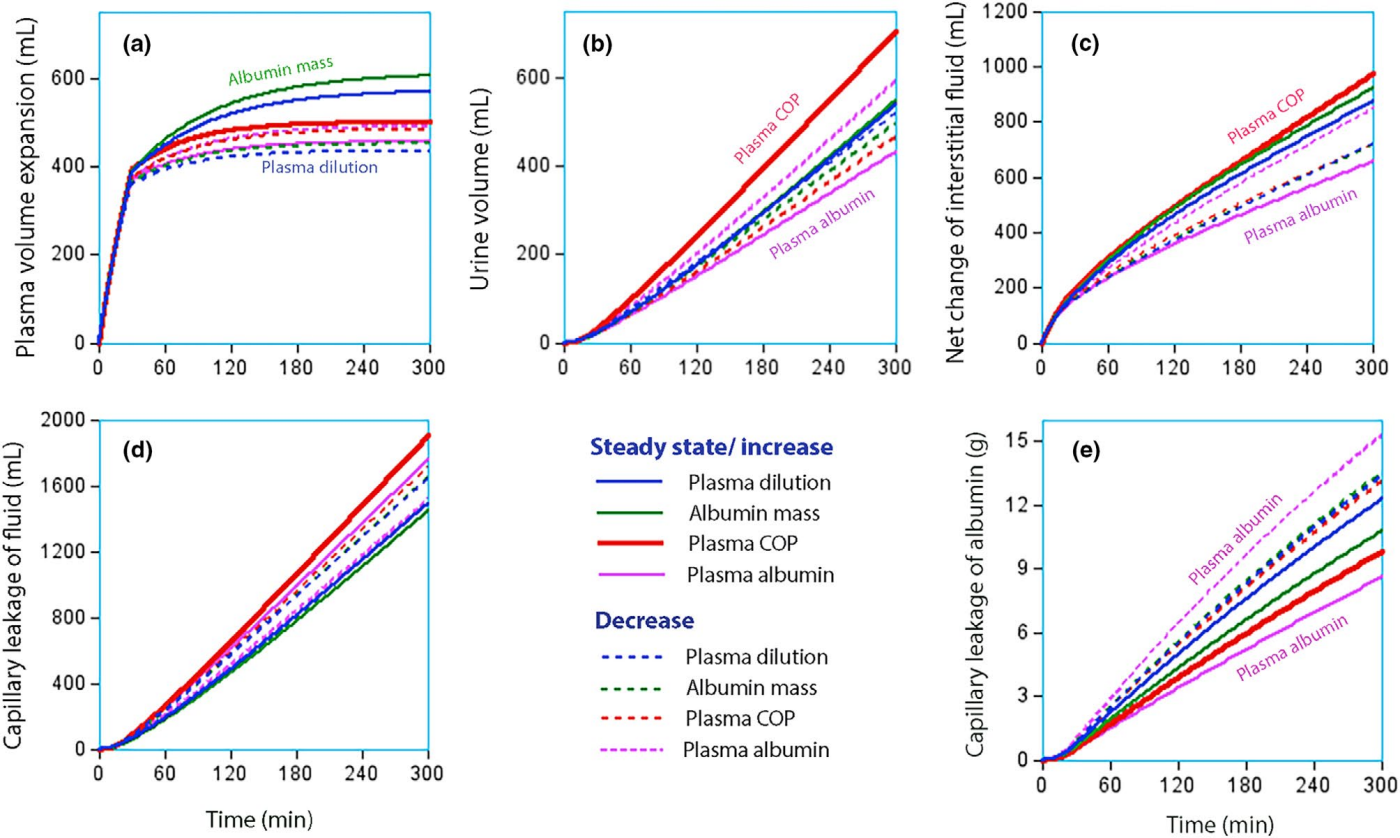
Simulations based on the “best estimate” of the kinetic parameters describing the fluid volume distribution depending on whether the concentration‐time plot showed steady state/increase (solid lines) or a decrease (dotted lines). (a) Volume expansion of Vc (central volume). (b) Urine output. (c) “Net change of interstitial fluid” is the difference between the oncotic‐driven shift of interstitial fluid to the plasma and the capillary leakage. Positive values imply dehydration. (d) Modeled capillary leakage of albumin molecules.

Simulations based on the kinetic parameters in Table 3 are shown in Figure 5. In general, SS curves tended to indicate a greater expansion of the PV compared with the curves that show a gradual decrease (Figure 5a). They had no consistent influence on urine output (Figure 5b) but more strongly dehydrated the interstitial space (Figure 5c) and leaked albumin molecules more slowly (Figure 5d). A summary of the characteristics of the simulated curves is given in Table 6.

**TABLE 6:** Arbitrary grading of the flow rates that characterize steady state (SS) patterns in biochemical variables over a period of 5 h after administration of 20% albumin. Symbols reflect the relative differences between the groups as shown in Figure 5.

Variable	Oncotic fluid shift	Urine output	Capillary leakage fluid	Capillary leakage albumin
SS for plasma dilution	0	0	−	0
SS for albumin mass	−	0	−	0
SS for plasma COP	++	++	++	−
SS for plasma albumin	0	−	+	−

*Note*: ++ high rate; + intermediate rate; 0 average rate; − low rate.

## 4 DISCUSSION

### 4.1 Key findings

Plasma dilution, COP, albumin, and the infused albumin mass often did not decrease as expected during a 5‐h follow‐up after infusion of 20% albumin. Deviations could sometimes be clearly associated with the body's handling of administered fluid and albumin molecules.

A key finding was that SS or an upward trend for plasma COP was associated with an overall faster turnover of fluid. This acceleration included faster capillary leakage of fluid and higher urine flow (Figure 5b,d). Despite the faster capillary leakage, the total effect on the interstitial volume was still that of dehydration (Figure 5c), which could possibly result in hyper‐oncotic anuria if aggravated by the administration of more 20% albumin. SS for plasma dilution and albumin mass were both associated with slow capillary leakage of fluid, and SS for the plasma albumin concentration also with slow albumin leakage (Table 5).

Overall, only modest differences in fluid and albumin distribution were usually required to account for deviating concentration–time curves.

### 4.2 Plasma dilution and fluid overload

Persistent plasma dilution is clinically important because it might predispose one to intravascular volume overload. This pattern was associated with slow fluid turnover, which statistically occurred more often during surgery. MAP was also lower, which might have contributed to the slightly lower *k*_10_ for urine flow (Hahn et al., 2024).

It might seem paradoxical that SS for dilution can occur despite a negative fluid balance; here, between two and three times more fluid was excreted than infused during the 5‐h study period. The explanation is that each gram of albumin binds 11 mL of water (Lamke & Liljedahl, 1976) and this oncotic strength translocates interstitial water to the plasma when albumin is administered in a hyper‐oncotic concentration. The fluid shift, which primarily occurs via the lymphatic route (Zdolsek et al., 2023), appeared to be quite consistent between the subgroups (*k*_21_ showed low variability), although the flow was accelerated by a large increase in plasma COP. This oncotic‐driven transfer of fluid is a continuous process and is unlikely to stop before the increased plasma albumin concentration has equilibrated with the interstitial fluid.

### 4.3 Plasma COP and kidney failure

Increasing plasma COP has been associated with the development of oliguria/anuria. The risk of oliguria/anuria and osmotic nephrosis associated with the administration of hyper‐oncotic colloid has been known for at least 60 years. Reported cases all show that very large amounts of hyper‐oncotic fluid have been infused.

Rozic & Paul (Rozich & Paul, 1989) described a 70‐year‐old man who developed anuria after being given 1800 g of 20% albumin over 72 h to mobilize ascites. P‐albumin was 118 g/L and COP was estimated to be 90 mmHg when anuria developed, but plasma creatinine had already begun to increase when plasma COP reached 40 mmHg. In volunteers, a trend toward reduced glomerular filtration has been found after increasing COP by as little as 3.5 mm by infusing 300 mL of albumin 20% (Boer et al., 1987). In the present study, the elevation of COP was greater, but usually for a short time only.

Ferraboli et al. (Ferraboli et al., 1997) reported two cases of renal failure after receiving 100 g of 10% dextran 40, which is another hyper‐oncotic colloid fluid. One liter of the fluid was given daily for 6 days. Each infusion is likely to increase the PV by at least 2 L (Hahn, 1988). Serum osmolality was 321 mosmol/kg when anuria developed, suggesting severe hyper‐oncoticity. Renal biopsy showed tubular swelling but neither glomerular alteration nor acute tubular necrosis.

Moran & Kapsner (1987) described a patient who became anuric after receiving 800 g of dextran 40 over 3 days after surgery. Plasma COP during anuria was 33 mmHg (20–25 mmHg is normal).

All patients described above recovered after plasmapheresis.

A non‐randomized study of 1013 patients reported a sixfold increase in the risk of anuria when 20% albumin was used to reverse hemodynamic instability in patients receiving intensive care (Schortgen et al., 2008), which might support that the renal sensitivity for hyper‐oncoticity is greater in sodium‐depleted and hypovolemic states (Boer et al., 1987). However, the validity of this clinical study has been debated (Wiedermann, 2009). The ALBIOS trial reported the same incidence of AKI for albumin 20% as for crystalloid fluid in septic patients (Caironi et al., 2014). Three literature reviews also found no increased incidence of AKI after treatment with albumin 20% compared with crystalloid fluid (Jacob et al., 2008; Jakob, 2004; Wiedermann et al., 2010).

This result of the present study suggests that hyper‐oncotic anuria must develop as a two‐stage process where the initial brisk urine flow reported here is later replaced by low flow due to volume depletion.

### 4.4 Kinetic analysis

The fluid and albumin kinetics were analyzed using population pharmacokinetics using log‐likelihood mathematics, which is a conventional tool to evaluate drug therapy. Models intended to mimic the known physiology were fitted to the data.

An issue with the fluid model is that the capillary leakage is not connected with the extravascular pool from where high plasma COP attracts fluid. However, the curve‐fitting became much poorer when connecting them, even when a time delay for the transfer was applied, which suggests that fluid that leaves the plasma by transcapillary filtration is not immediately available for recruitment by hyper‐oncotic plasma.

Another issue is that Figure 5 does not consider COP variations over time. Ideally, new flow values should be provided every time plasma dilution is measured. The increase in COP was greatest at the end of the infusions, and therefore, Figure 5a should have a slightly different curvilinear appearance (Hahn et al., 2019).

The two groups with inflammation (post‐burn and postoperative) had lower plasma albumin and plasma COP at baseline (Table 1). The covariance analysis showed that inflammation was associated with slower elimination of fluid and a smaller distribution volume for the albumin mass. These influences on the kinetics were stronger in the postoperative than the post‐burn setting, as “postoperative” also served as an independent covariate in the analysis of both the fluid and albumin mass kinetics. Inclusion of the baseline plasma albumin and plasma COP did not improve the model in addition to these groupwise influences.

The distribution of deviating curves between the subgroups is naturally affected by the modeled average half‐lives reported for the respective subgroups. Specifically, this was an issue for the curves derived during surgery, where the turnover was slow. However, there may be many physiological reasons for why the kinetic parameters differed between the subjects. Accelerated capillary leakage of albumin and fluid can be due to damage to the endothelial glycocalyx layer (Rehm et al., 2007). However, the plasma concentration of syndecan‐1, which is a surrogate measure of glycocalyx injury, did not indicate increased capillary leakage in the post‐burn patients (Hahn et al., 2021). The urinary excretion differed between the clinical settings (Figure 3c), but it also correlated with the urinary creatinine concentration before the experiment started (Figure 3d), which is, in turn, governed by the habitual ingestion of water during the days preceding the experiment (Hahn, 2021).

### 4.5 Clinical implications

Albumin 20% is a potent PV expander with a prolonged effect. The PV is expanded by twice the infused volume which, in most cases, is only reduced by 50% the next day. SS for plasma dilution is quite common in settings with fluid retention and, therefore, caution with repeated infusions of albumin 20% in clinical situations with fluid retention is warranted.

The association between fast turnover of fluid and hyper‐oncoticity questions the practice of dehydrating patients with edema by combining 20% albumin with furosemide injections, at least before the diuretic response to the albumin infusion has been evaluated.

The most important precaution is to keep the infused volume low. The present experiments used 200 mL and were not followed by symptoms or undue consequences. However, the same additional amount administered a few hours later, or the next day, could cause clinical problems.

The published case reports show that excessive amounts of hyper‐oncotic fluid are needed for oliguria/anuria to develop (Ferraboli et al., 1997; Moran & Kapsner, 1987; Rozich & Paul, 1989). This complication does not seem to cause persistent kidney injury but requires plasmapheresis to resolve. Oliguria/anuria is unlikely to occur with a reasonably restrictive policy, and population studies hold that modern use of albumin 20% is safe (Caironi et al., 2014; Jacob et al., 2008; Wiedermann et al., 2010).

### 4.6 Limitations

Limitations include that the author's subjective judgment was used to decide whether SS occurred during the 5‐h period of the study. This ultimately results in classifications that can be debated. They are shown together with the underlying data in File S2. However, using a strict rule for how to grade the concentration‐time curves did not seem adequate. Most curves with “gradual decrease” fell by 25% or more during the 5‐h follow‐up period, but curves could also show a consistent decrease while occurring more slowly. The concentration‐time curves occasionally showed an upward or unclear trend during the first 2 hours post‐infusion and then a clear gradual decrease, and these curves were classified as “gradual decrease” regardless of the decrease rate or the direction of the regression line. Curves that showed no clear trend were left unclassified.

It is not certain that the patterns analyzed here can be considered aberrant or would even be the same if studied after 24 h or 48 h. The kinetics still suggest that the findings are results of an imbalance between the distribution rates and elimination of fluid and albumin that may predispose to fluid overload and hyper‐oncotic anuria if aggravated.

It might be surprising that SS for plasma dilution and plasma albumin were not significantly associated. However, plasma dilution is governed by an interplay between the three fluid kinetic parameters (*k*_12_, *k*_b_, and *k*_21_), while the plasma albumin concentration is also affected by the albumin kinetics. As one might expect, SS for plasma albumin was associated with a quite slow capillary leakage rate of albumin molecules (Table 5).

The study did not include severely ill patients but volunteers, post‐burn patients, and patients undergoing lengthy but not greatly invasive surgery. Therefore, the results should be regarded as characteristics of 20% albumin without the influence of acute disease.

Norepinephrine was used in <10% of the subjects. An analysis of its influence on the kinetics was made in the original publication and consisted of the acceleration of the urine flow and capillary leakage fluid. No effect on the intravascular albumin mass was found (Gunnström et al., 2022).

The modeled central volume (*V*_c_) for fluid agreed well with Nadler's anthropometric equations. Kinetic parameters derived post hoc must be used for such comparisons as the *V*_c_ reported in Tables 3 and 4 is not influenced by covariates at all. However, *V*_c_ for the albumin mass was still much larger, which might be due to immediate partial extravascular distribution of albumin. The discrepancy between the distribution volumes for circulating plasma and albumin molecules is denoted the “f‐cell ratio” and is reported to vary between 5% and 15% (mean 9%) (Chaplin Jr et al., 1953) although it can increase during hemodilution (Haller et al., 1995). The explanation is probably that albumin freely enters the liver sinusoids, while the firm liver capsule prevents volume expansion by fluid (Hahn, 2024). In the present study, the difference is greater than 9%, but the kinetic analysis is not focused only on this parameter but strives to keep the total error for the model as low as possible. Another contributing factor could be that the fluid model is simply more accurate than the albumin mass model.

The used data have been published in four different studies over a period of 4 years, albeit never reported from the present standpoint.

The kinetic constants express changes in flow rates induced by the infusion of 20% albumin. The baseline rates are not included.

A strength of this study is that all experiments were performed in a similar way, using the same protocol, and by the same researchers.

## 5 CONCLUSIONS

Deviations of washout curves after infusion of albumin 20% were common. SS for plasma dilution corresponded to slow turnover of fluid, while SS or increasing plasma COP corresponded to fast turnover of fluid. Albumin 20% has an intrinsic diuretic effect and, to avoid hyper‐oncoticity, the diuretic response should be evaluated before another diuretic is administered. Hyper‐oncotic albumin is a potent plasma volume expander, and caution with repeated doses is warranted.

## AUTHOR CONTRIBUTIONS

RGH planned and supervised all the included studies. RGH also initiated the current analysis, made the calculations, and authored the manuscript.

## FUNDING INFORMATION

No specific funding was given for the present study.

## CONFLICT OF INTEREST STATEMENT

RGH has received a research grant from Grifols for a study of 20% albumin for goal‐directed fluid therapy during surgery.

## ETHICS STATEMENT

The studies were performed in accordance with relevant guidelines and regulations. They were approved by the Regional Ethics Committee of Linköping, Sweden (Dnr 2017/478–31 and 2016/333–32) and by the Regional Ethics Committee in Stockholm, Sweden (Nr. 2014/2146–31/1). Database registrations were clinicaltrials.gov NCT02952378, NCT02556580, EudraCT 2016‐000996‐26, and Eudra‐CT 2017‐003687‐12. All database registrations were made before any subject was enrolled. Written informed consent was obtained from all participants before any experiment was initiated.

## Supporting information


File S1.

File S2.

## Data Availability

The original data are given in File S2.
