# Supplementary material for: Constant plasma volume and colloid osmotic pressure after infusion of albumin 20%: A secondary analysis
Source: Physiol Rep. 2025 Oct 22;13(20):e70623. doi: 10.14814/phy2.70623 (PMC12546676; doi:10.14814/phy2.70623)
Supplement: Supplementary file 1 — File S1. [file PHY2-13-e70623-s001.pdf]

Supplementary File 1

Fluid kinetic model

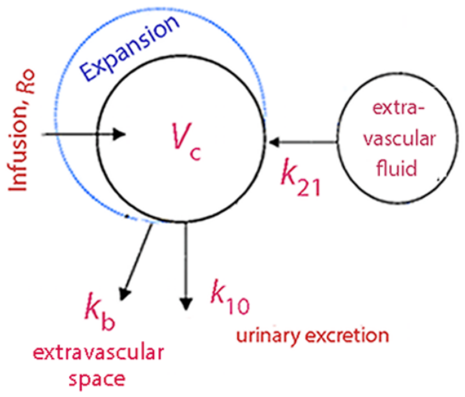

Fluid is infused at the rate R_o into the plasma (V_c) which then becomes expanded (v_c). Distribution to the extravascular space occurs at a rate determined by a constant k_b and as urine (U) at rate governed by the constant k_{10}. Absorption of fluid to V_c from the extravascular space is governed by a rate constant k_{21} and it is assumed the available fluid pool is the interstitial fluid volume (IFV), which is 15% of the body weight.

The flow rate is the product of the rate constant describing the flow and the volume expansion of V_c at the same point in time, the exception being k_{21} which is the exponent in a mono-exponential absorption function. The differential equations describing the model are the following:

$$dv_c/dt = R_o - k_b (v_c - V_c) - k_{10} (v_c - V_c) + k_{21}\,\mathrm{IFV}$$

$$d\mathrm{IFV}/dt = - k_{21}\,\mathrm{IFV_o}$$

$$dU/dt = k_{10} (v_c - V_c)$$

The term k_{21} IFV does not operate at baseline but begins when the infusion of 20% albumin starts. The plasma dilution corresponds to $(v_c - V_c) / V_c$. The excreted urine, whenever collected, was used as input variable for U.

Covariance analyses

The fixed rate constants k_b, k_{21}, and k_{10} and the size of V_c could all be modified by *covariates*, which are characteristics that may change the parameter estimates in an individual or in a group. The most promising candidates for covariate effects was searched by plots of random effects ("eta:s"). These variables were then added one by one to the model and accepted for inclusion if the -2 log likelihood (-2 LL) for the model then decreased by > 3.84 points ($P < 0.05$).

Three covariate models were used. The **power model** was applied for continuous variables. Here, the only variable for which this covariance model is suitable was the relationship between urine creatinine and the rate constant for urine flow, k_{10} (see Table 3). The fixed parameter value was 0.00575, the covariate value -0.30, and the mean urine creatinine concentration at baseline was 12.6 mmol/L. The k_{10} for a person with urine creatinine of 5 mmol/L before the infusion starts then becomes

$$k_{10} = 0.00575 * [(5/\ 12.6)^{-0.30}] = 0.00759$$

The urine flow rate is 32% higher when urine creatinine is 5 mmol/L compared to if it would have the mean value for the group of 12.6 mmol/L.

The **exponential model** is used for categorical variables. For example, the size of V_c was lower by female sex. The group value for V_c of 3,937 mL then represents only the males, while the size of V_c for the females is obtained using the natural logarithm e = 2.718 and the covariance factor -0.31 as follows:

$$V_c = 3{,}937\ [e^{-0.31}] = 2{,}887$$

This means that females had a size of V_c that was approximately 1 L smaller than the males.

The third covariance model is the **linear model** which is used when continuous values may be zero or negative. The example in the present study is the change in plasma oncotic pressure from baseline. This variable affected k_{21}, which had the group value of 0.00186, which corresponded to the average change in colloid pressure of 2.145 (for all measurements of of the collod pressure in the study). The covariate strength was0.34 If the change in pressure is 6 mmHg, the value of k_{21} becomes:

$$k_{21} = 0.00186 * (1 + 0.34\ (6 - 2.145))$$

This covariance is time-dependent, i.e., a new value for k_{21} is calculated for each point in time, which differs from the covariates why imply that the same change of the fixed covariate persists throughout the experiment.

Albumin kinetic model

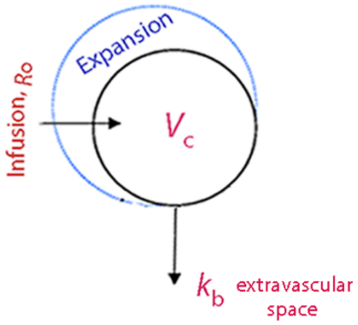

The kinetics of the administered albumin mass was analyzed by using a one-volume model. The infused albumin is infused at the rate R_o into the plasma volume (V_c) from where the albumin is eliminated by capillary leakage (k_b). The P-albumin concentration gives a false impression of the albumin mass that is present in V_c because of the volume expansion of V_c to v_c.. Therefore, the differential equation for the change in intravascular mass (dX) becomes:

$$dX / dt = R_o - k_b [(\Delta P\text{-albumin}_{t-0} * (1 + P\text{-dilution}_t)]$$

Covariance analysis was performed to refine the analysis of the infused albumin mass, too, just as was done for the fluid model. The final parameter estimates are given in Table 4.

Theta	Estimate	LogLik	RetCode	Delta	Percent
tvV	3935.8271	1057.4695	3		0
tvV	4722.9926	1055.3073	3		20
tvKe	0.0057518033	1057.4683	3		0
tvKe	0.0069021639	1055.9275	3		20
tvkb	0.039206396	1057.4696	3		0
tvkb	0.047047675	1053.7052	3		20
tvkabs	0.0018569539	1057.4703	3		0
tvkabs	0.0022283446	1052.0214	3		20
dKedInflammati	-0.31356647	1057.4683	3		0
dKedInflammati	-0.37627977	1057.4007	3		20
dVdGenderMale	-0.30706603	1057.4703	3		0
dVdGenderMale	-0.36847924	1057.2841	3		20
dKedUcrea	-0.29781943	1057.4682	3		0
dKedUcrea	-0.35738332	1057.3074	3		20
dVdCOPdiff	0.090241056	1057.4682	3		0
dVdCOPdiff	0.10828927	1054.6022	3		20
dkbdPostop1	-0.18309936	1057.4682	3		0
dkbdPostop1	-0.21971923	1057.3655	3		20
dKedPostop1	-0.36237293	1057.4682	3		0
dKedPostop1	-0.43484752	1057.4407	3		20
dkbdCOPdiff	0.35897824	1057.4681	3		0
dkbdCOPdiff	0.43077389	1044.8159	3		20
dkabsdCOPdiff	0.33999248	1057.4694	3		0
dkabsdCOPdiff	0.40799097	794.4853	1		20

Sensitivity analysis for the fluid kinetic model. Perturbation was set to 20% of the variables shown in Table 3. This perturbation resulted in only small changes of the -2 LL except for the last covariate, which is the onfluence of the COP on the rate pf fluid transfer from the interstitium to the plasma (k_{21}). The return code 3 means that the last step in the iteration failed to fund a better solution than the reported one. Pasted from the Phoenix output.

Theta	Estimate	LogLik	RetCode	Delta	Percent
tvV	5.7519051	-2047.2442	3		0
tvV	8.6278577	-2103.4033	1		50
tvkb	0.00083137129	-2047.2442	3		0
tvkb	0.0012470569	-2052.8629	1		50
dVdPostop1	-0.033374786	-2047.2441	3		0
dVdPostop1	-0.050062179	-2047.2733	1		50
dVdBodyweight	1.4779185	-2047.244	3		0
dVdBodyweight	2.2168777	-2055.6213	1		50
dVdBMI	-0.89371537	-2047.2441	3		0
dVdBMI	-1.3405731	-2050.0044	1		50
dVdUrine	-0.095409627	-2047.2441	3		0
dVdUrine	-0.14311444	-2048.3434	1		50
dkbdGenderMal	0.61223196	-2047.2441	3		0
dkbdGenderMal	0.91834793	-2049.1078	1		50
dVdInflammatio	-0.14486533	-2047.2441	3		0
dVdInflammatio	-0.21729799	-2048.3346	1		50
dkbdBMI	-1.0005797	-2047.2442	3		0
dkbdBMI	-1.5008696	-2047.8383	1		50

Sensitivity analysis for the kinetic model for albumin mass. Perturbation was set to 50% of the variables shown in Table 5. Here, the value was set higher because 20% created virtually no changes of -2 LL. The return code 1 means that the relative gradient is close to zero and that the reported solution is likely to be optimal or nearly optimal. Pasted from the Phoenix output.

PROGRAM FILE FOR THE ANALYSIS OF FLUID VOLUME

```
test(){
        deriv(A1 = - (A1 * Ke)- (A1 * kb) + (Aa * k21))
        urinecpt(A0 = (A1 * Ke))
        urinecpt(A2 = (A1 * kb))
        deriv(Aa = - (Aa * k21)+(A1 * kb))
        C = A1 / V
        dosepoint(A1, idosevar = A1Dose, infdosevar = A1InfDose, infratevar = A1InfRate)
        error(CEps = 0.0280961344384084)
        observe(CObs = C + CEps)
        error(A0Eps = 150.421476382701)
        observe(A0Obs = A0 + A0Eps)
        dosepoint(Aa, idosevar = AaDose, infdosevar = AaInfDose, infratevar = AaInfRate)
        stparm(V = tvV * exp(nV))
        stparm(Ke = tvKe * exp(nKe))
        stparm(kb = tvkb * exp(nkb))
        stparm(k21= tvk21* exp(nkabs))
        fixef(tvV = c(,2309.00093964,))
        fixef(tvKe = c(,0.00768040779255089,))
        fixef(tvkb = c(,0.0100025653665529,))
        fixef(tvkabs = c(,0.000408395582155461,))
        ranef(diag(nKe, nkb, nk21, nV) = c(0.4729288, 0.3510482, 1.3799226E-11, 0.20007977))
}
```

PROGRAM FILE FOR THE ANALYSIS OF MASS

```
test(){
        deriv(A1 = - (A1 * kb))
        urinecpt(A2 = (A1 * kb))
        C = A1 / V
        dosepoint(A1, idosevar = A1Dose, infdosevar = A1InfDose, infratevar = A1InfRate)
        error(CEps = 1.1762277857198)
        observe(CObs = C + CEps)
        stparm(V = tvV * exp(nV))
        stparm(kb = tvkb * exp(nkb))
        fcovariate(Inflammation())
        fcovariate(Operation())
        fcovariate(Postop())
        fcovariate(Vol0Post1Op2Burn3())
        fcovariate(Age)
        fcovariate(GenderMale0())
        fcovariate(Bodyweight)
        fcovariate(Pkreatinin)
        fcovariate(MAP)
        fcovariate(Albumin0)
        fcovariate(Fluidtype())
        fixef(tvV = c(, 5.46363794037803, ))
        fixef(tvkb = c(, 0.00116991774458751, ))
        ranef(diag(nkb, nV) = c(0.31958416, 0.072648935))
```
